# Draft genome of *Brasenia schreberi*, a worldwide distributed and endangered aquatic plant

**DOI:** 10.1186/s12863-024-01212-2

**Published:** 2024-03-04

**Authors:** Lin-Fang Wu, Wei-Guang Zhu, En-Ping Yu, Hong-Lin Cao, Zheng-Feng Wang

**Affiliations:** 1 Guangzhou Linfang Ecological Technology Co., Ltd, 510000 Guangzhou, China; 2 Key Laboratory of Vegetation Restoration and Management of Degraded Ecosystems, South China Botanical Garden, Chinese Academy of Sciences, 510650 Guangzhou, China; 3 Key Laboratory of National Forestry and Grassland Administration on Plant Conservation and Utilization in Southern China, South China Botanical Garden, Chinese Academy of Sciences, 510650 Guangzhou, China; 4 Guangdong Provincial Key Laboratory of Applied Botany, South China Botanical Garden, Chinese Academy of Sciences, 510650 Guangzhou, China; 5 South China National Botanical Garden, 510650 Guangzhou, China; 6 University of Chinese Academy of Sciences, 100049 Beijing, China

**Keywords:** Gene prediction, Genome feature, Genome annotation, Repetitive sequences

## Abstract

**Objectives:**

*Brasenia* is a monotypic genus in the family of Cabombaceae. The only species, *B*. *schreberi*, is a macrophyte distributed worldwide. Because it requires good water quality, it is endangered in China and other countries due to the deterioration of aquatic habitats. The young leaves and stems of *B*. *schreberi* are covered by thick mucilage, which has high medical value. As an allelopathic aquatic plant, it can also be used in the management of aquatic weeds. Here, we present its assembled and annotated genome to help shed light on medial and allelopathic substrates and facilitate their conservation.

**Data description:**

Genomic DNA and RNA extracted from *B*. *schreberi* leaf tissues were used for whole genome and RNA sequencing using a Nanopore and/or MGI sequencer. The assembly was 1,055,148,839 bp in length, with 92 contigs and an N50 of 22,379,495 bp. The repetitive elements in the assembly were 555,442,205 bp. A completeness assessment of the assembly with BUSCO and compleasm indicated 88.4 and 90.9% completeness in the Eudicots database and 95.4 and 96.6% completeness in the Embryphyta database. Gene annotation revealed 67,747 genes that coded for 73,344 proteins.

## Objective

*Brasenia schreberi* is an aquatic and perennial herb in the Cabombaceae family. It is a monotypic species with oval-shaped leaves that can submerge or float on the water’s surface, similar to water lilies. Except for Europe and Antarctica, it is currently distributed on all continents of the world [1]. However, palaeobotanical records indicate that *B*. *schreberi* was a frequent element in Europe before the last glacial period [1]. Its habitats include ponds, lakes, and sluggish streams, but they must be clean and acidic and have nutrient-enriched sediment [1, 2]. Due to the deterioration of water quality and habitat loss, it is listed at the second level of national key protected wild plants in China and is endangered in other countries [2, 3]. Its edible young leaves and stems are coated with a thick mucilage that is mainly composed of polysaccharides and has high medical value [2, 4, 5]. Mucilage has been confirmed to be a defense against herbivores and bacteria [3, 6]. *Brasenia schreberi* contains allelopathic components that can be used in the management of aquatic weeds [7]. As important values in taxonomy, ecology, and economy and in its endangered situation, a genome assembly was published previously [8] for its better conservation and breeding. However, given its wide distribution worldwide and existing substantial genetic diversities [3, 9], we present an alternative *B*. *schreberi* genome to better understand its evolution and adaptation and to enhance its conservation, management, and utility in the future.

## Data description

Leaf samples of *B*. *schreberi* were collected from an individual planted in the South China Botanical Garden, Guangzhou, China. The DNA or RNA extracted from its leaf tissues was used to construct three sequencing libraries, including long read whole genome sequencing (WGS) using a Nanopore PromethION sequencer, short read WGS using an MGI DNBSEQ-T7 sequencer, and RNA sequencing (RNA-seq) using an MGI DNBSEQ-T7 sequencer. Under the MGI platforms, a 150 bp paired-end mode was applied for both short WGS and RNA-seq. The long-read WGS generated about 113.0 GB of data (Data file 1) [10], short-read WGS generated about 130.6 GB data (Data file 2) [11], and RNA-seq generated about 27.6 GB data (Data file 3) [12].

After sequencing, short WGS reads were trimmed by Sickle v1.33 [13] using the parameter “-q 30 -l 80”. KmerGenie v1.7044 [14] (under the parameter of “-k 141 --diploid”) was then used to estimate the genome size of *B*. *schreberi* with trimmed short WGS reads. The estimated genome size was 963,304,542 bp. Porchop v0.2.4 [15] and ontbc v1.1 [16] were used to remove adapter and low-quality reads (scores < 7 and lengths < 5000 bp) in long WGS reads. NextDenovo v2.3.1 [17] was then used to assemble the genome with the filtered long reads. Pseudohaploid [18] and Purge_Dups v1.2.6 [19] were applied to remove redundant contigs. Subsequently, Racon v1.5.0 [20], hapo-G v1.3.2 [21], and polypolish v0.5.0 [22] were used to polish the assembly. The final assembly was 1,055,148,839 bp in length, with 92 contigs and a contig N50 of 22,379,495 bp (Data file 4) [23]. BUSCO v5.5.0 [24] and compleasm v0.2.5 [25] were used to assess the completeness of the assembly with Eudicots odb10-2020-09-10 and Embryphyta odb10 2020-09-10 databases. BUSCO revealed 88.4 and 95.4% completeness in the Eudicots and Embryphyta databases, respectively (Data files 5–6) [26, 27]. Compleasm revealed 90.9 and 96.7% completeness in the Eudicots and Embryphyta databases, respectively (Data files 7–8) [28, 29].

Repetitive elements in the *B*. *schreberi* assembly were estimated by RED v2.0 [30] and EDTA v2.1.3 [31], which revealed 452,408,938 (Data file 8) [32] and 521,424,853 bp (Data file 9) [33] of sequences, respectively. Combining the RED and EDTA results revealed 555,442,205 bp of repetitive sequences (Data file 10) [34], which were used to soft-mask the assembly. Braker3 v.3.0.6 [35] was used to predict the primary gene structures using transcriptome data and reference protein sequences (Data file 11) [36]. The Braker results were then incorporated into the Funannotate pipeline v1.8.16 [37] to obtain integrated gene sets. The pipeline included four steps: “train”, “predict”, “update”, and “annotate”. For the former three steps, the parameter “--max_intronlen 1000000” was used, while in the “predict” step, the parameters “--busco_seed_species arabidopsis --organism other --busco_db embryophyta” were added. The fourth “annotate” step was used for gene function annotation. The final gene prediction obtained 67,747 protein-coding genes and 813 tRNA genes (Data files 12–14) [38–40]. Functional annotation of protein-coding genes is shown in Data files 15–16 [41, 42].

## Limitations

The current *B*. *schreberi* assembly in this study is fragmented. Future sequencing technologies, including Hi-C, Nanopore ultra-long sequencing, PacBio HiFi, 10X Genomics linked sequencing, and Bionano optical maps, are needed for complete and gapless genome assembly.

However, our assembly displayed a completeness comparable to the previously reported *B*. *schreberi* assembly [8], which showed 89.0 and 95.9% completeness using BUSCO in the Eudicots and Embryphyta databases, respectively, and 91.3% and 97.0% completeness using compleasm in the Eudicots and Embryphyta databases, respectively. Nevertheless, because this previous assembly did not remove duplications from the assembly [43], some assembly errors may exist for gene prediction. For the completed BUSCOs, our assembly revealed 39.7% and 46.8% higher complete and single-copy BUSCOs using BUSCO in the Eudicots and Embryphyta database, while it was 37.4 and 44.0% complete and single-copy BUSCOs in Eudicots and Embryphyta for the previously reported assembly. Using compleasm, our assembly was shown to have 47.9 and 54.7% complete and single-copy BUSCOs in the Eudicots and Embryphyta database, while it was 44.54 and 50.9% complete and single-copy BUSCOs in the Eudicots and Embryphyta database for the previously reported assembly. Therefore, our assembly contained a few duplication errors in the assembly for better gene prediction.

**Table 1:** Overview of all data files/data sets

Label	Name of data file/data set	File types (file extension)	Data repository and identifier (DOI or accession number)
Data file 1	Raw long whole genome sequencing reads	Fastq file (.fastq)	NCBI Sequence Read Archive, https://identifiers.org/ncbi/insdc.sra:SRR27392947 [10]
Data file 2	Raw short whole genome sequencing reads	Fastq file (.fastq)	NCBI Sequence Read Archive, https://identifiers.org/ncbi/insdc.sra:SRR27392979 [11]
Data file 3	Raw RNA reads of leaf tissues	Fastq file (.fastq)	NCBI Sequence Read Archive, https://identifiers.org/ncbi/insdc.sra:SRR27392978 [12]
Data file 4	Assembled genome	Fasta file (.fasta)	NCBI Nucleotide, https://identifiers.org/nucleotide:JAYKKT000000000 [23]
Data file 5	BUSCO assessment of the assembly using Eudicots database	Text (.txt)	Figshare, 10.6084/m9.figshare.25036598 [26]
Data file 6	BUSCO assessment of the assembly using Embryphyta database	Text (.txt)	Figshare, 10.6084/m9.figshare.25036601 [27]
Data file 7	Compleasm assessment of the assembly using Eudicots database	Text (.txt)	Figshare, 10.6084/m9.figshare.25036607 [28]
Data file 8	Compleasm assessment of the assembly using Embryphyta database	Text (.txt)	Figshare, 10.6084/m9.figshare.25036613 [29]
Data file 8	Repetitive sequences predicted by RED	Text file (.bed)	Figshare, 10.6084/m9.figshare.25092815.v1 [32]
Data file 9	Repetitive sequences predicted by EDTA	Gff3 file (.gff3)	Figshare, 10.6084/m9.figshare.25092836.v1 [33]
Data file 10	Repetitive sequences combined by RED and EDTA	Text file (.bed)	Figshare, 10.6084/m9.figshare.25092983.v1 [34]
Data file 11	Table 1 Species with their protein sequences used for gene prediction	Table (.xlsx)	Figshare, 10.6084/m9.figshare.25093304.v1 [36]
Data file 12	Predicted gene	Gff3 file (.gff3)	Figshare, 10.6084/m9.figshare.25093376.v2 [38]
Data file 13	Predicted genes - nucleotide sequences	Fasta file (.fasta)	Figshare, 10.6084/m9.figshare.25093382.v1 [39]
Data file 14	Predicted genes - translated sequences	Fasta file (.fasta)	Figshare, 10.6084/m9.figshare.25093385.v1 [40]
Data file 15	Gene annotation using GO, Pfam, interPro, UniProt, dbCAN, MEROPS and SignalP databases	Text (.txt)	Figshare, 10.6084/m9.figshare.25093394.v1 [41]
Data file 16	Gene annotation from eggNOG-mapper analysis	Text (.txt)	Figshare, 10.6084/m9.figshare.25093397.v1 [42]

## Data Availability

Raw sequenced reads have been uploaded to the NCBI Sequence Read Archive under accession number SRR27392947 for long whole genome sequencing reads [10], SRR27392979 for short whole genome sequencing reads [11], SRR27392978 for RNA-seq reads [12], and JAYKKT000000000 for the assembled genome [13]. Please further see Table 1 for details and references [32-34,36,38-42] of the results of the annotations submitted to figshare.
